# Novel Method for Estimating Propulsive Force Generated by Swimmers’ Hands Using Inertial Measurement Units and Pressure Sensors

**DOI:** 10.3390/s22176695

**Published:** 2022-09-04

**Authors:** Tomoya Kadi, Tomohito Wada, Kenzo Narita, Takaaki Tsunokawa, Hirotoshi Mankyu, Hiroyuki Tamaki, Futoshi Ogita

**Affiliations:** 1 Graduate School of Physical Education, National Institute of Fitness and Sports in Kanoya, Kanoya 891-2393, Japan; 2 Human Augmentation Research Center, National Institute of Advanced Industrial Science and Technology, Kashiwa 277-0882, Japan; 3 Information Technology Center for Sports Sciences, National Institute of Fitness and Sports in Kanoya, Kanoya 891-2393, Japan; 4 Faculty of Sports and Budo Coaching Studies, National Institute of Fitness and Sports in Kanoya, Kanoya 891-2393, Japan; 5 Advanced Research Initiative for Human High Performance (ARIHHP), Faculty of Health and Sport Sciences, University of Tsukuba, Tsukuba 305-8574, Japan; 6 Faculty of Sports and Life Science, National Institute of Fitness and Sports in Kanoya, Kanoya 891-2393, Japan

**Keywords:** human swimming, underwater motion-capture system, front crawl swimming, accelerometer, gyroscope

## Abstract

Propulsive force is a determinant of swimming performance. Several methods have been proposed to estimate the propulsive force in human swimming; however, their practical use in coaching is limited. Herein, we propose a novel method for estimating the propulsive force generated by swimmers’ hands using an inertial measurement unit (IMU) and pressure sensors. In Experiment 1, we use a hand model to examine the effect of a hand-mounted IMU on pressure around the hand model at several flow velocities and water flow directions. In Experiment 2, we compare the propulsive force estimated using the IMU and pressure sensors ($F_{\mathrm{IMU}}$) via an underwater motion-capture system and pressure sensors ($F_{\mathrm{Mocap}}$). Five swimmers had markers, pressure sensors, and IMUs attached to their hands and performed front crawl swimming for 25 m twice at each of nine different swimming speeds. The results show that the hand-mounted IMU affects the resultant force; however, the effect of the hand-mounted IMU varies with the flow direction. The mean values of $F_{\mathrm{Mocap}}$ and $F_{\mathrm{IMU}}$ are similar (19.59 ± 7.66 N and 19.36 ± 7.86 N, respectively; intraclass correlation coefficient_(2,1)_ = 0.966), and their waveforms are similar (coefficient of multiple correlation = 0.99). These results indicate that the IMU can estimate the same level of propulsive force as an underwater motion-capture system.

## 1. Introduction

Swimming is a physical exercise performed in water. Numerous studies have been conducted on human swimming from a mechanical perspective. Swimmers use their hands and legs to push water backward and propel their bodies forward. Swimmers generate propulsive force primarily using their hands during swimming, except for the breaststroke style. Since the propulsive force is a determinant of swimming velocity, the propulsive force must be evaluated to analyze the swimmer’s performance. To date, various methods for estimating the propulsive force have been proposed, such as underwater video analysis [1,2,3,4], force measurement in tethered swimming [5,6,7], and pushing a fixed plate submerged in water [8,9]. These studies pioneered methods for estimating the propulsive force of swimmers. However, many researchers have highlighted the importance of considering unsteady flow around swimmers [10,11,12,13,14], and these methods do not consider the effects of unsteady flow; thus, a different approach is required.

Takagi and Wilson [15] proposed a method of estimating the magnitude of hydrodynamic force acting on the hands of swimmers by attaching pressure sensors. Pressure sensors can obtain the pressure acting perpendicular to the plane of the pressure sensor. The authors multiplied the pressure difference between the palm and the dorsal sides of the hand by the hand area to obtain the hydrodynamic force. This method allows one to investigate the hydrodynamic force acting on the swimmer’s hands and its variations while considering unsteady flow. However, the direction of the hydrodynamic force cannot be determined using pressure sensors alone. The hand direction must be evaluated to determine the direction of the hydrodynamic force acting on the hand.

Recently, underwater motion-capture systems have been introduced for swimming research [16,17,18,19]. Tsunokawa et al. [18] used an underwater motion-capture system combined with pressure sensors to analyze the resultant force ($F_{R}$) acting on the hand and estimated the propulsive force. This method enables the estimation of propulsive force while considering unsteady flow; however, underwater motion-capture systems present several issues. For instance, when swimmers place their hands in water, bubbles are generated around them. The bubbles cause the diffusion and absorption of light, which result in reduced visibility of the reflective markers attached to their hands [16,17,20]. In addition, underwater motion-capture systems limit the measurement range and require considerable time for preparing and attaching reflective markers to the participants.

In recent years, the development of microelectromechanical systems technology has enabled sensor miniaturization. In addition, the development of waterproof technology has enabled the application of inertial measurement units (IMUs) with multiple sensors in swimming research [21,22,23,24,25,26,27,28,29,30]. Lanotte et al. [29] developed a system that provides information about the force and the swimmer’s motion, measured by combination of mini-paddles, built-in pressure sensors, and an IMU. In this early research, IMUs were used to observe motion with raw data obtained by the sensor. An IMU is generally equipped with accelerometers, gyroscopes, and magnetometers, and it can estimate its orientation via sensor fusion. Therefore, using an IMU does not cause issues arising from bubbles. Washino et al. [19] indicated that reflective markers attached to swimmers produced an additional drag force, which decreased the swimming speed. Motion-capture systems require multiple markers to be attached to a single segment to estimate the orientation of a segment. By contrast, a segment’s orientation can be estimated by mounting a single IMU on the segment, which may decrease the drag force produced by the measurement equipment. Moreover, IMUs enable the measurement of a wider range of areas than conventional video analysis. However, similar to reflective markers, IMUs may affect the water flow. The pressure acting on the hand changes with the water flow. The water flow around the hand will be different when an IMU is mounted on the hand, which may result in pressure differences. Therefore, the effect of IMUs on the pressure around the mounted area must be examined.

The purpose of this study is to propose a novel method for estimating the propulsive force generated by a swimmer’s hand using an IMU and pressure sensors. To achieve this goal, two experiments are conducted. The first experiment is conducted to verify whether the $F_{R}$ acting on the model with or without an IMU differs while the velocity and water flow direction to the model are changed. The second experiment is performed to compare the propulsive force estimated using an IMU and pressure sensors via an underwater motion-capture system and pressure sensors to evaluate the agreement between the two methods.

## 2. Materials and Methods

### 2.1. Experiment 1: Effect of Hand-Mounted IMU on Pressure around Hand Model

#### 2.1.1. Experimental Design

**Figure 1:**
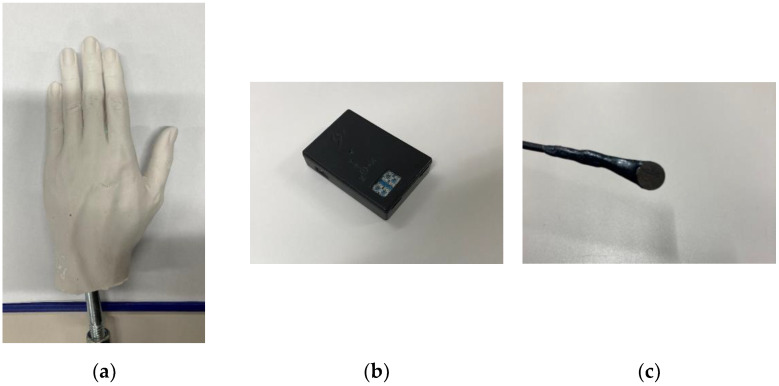
Hand model and sensors used in Experiment 1. (**a**) Silicone hand model measured 0.19 m × 0.095 m × 0.04 m (length × width × thickness). Projected area of hand model measured 0.014 m^2^. (**b**) Waterproof IMU measured 38 mm × 53 mm × 11 mm. IMU weighed 32 g. (**c**) Pressure sensor measured 0.6 mm in thickness and 6.0 mm in diameter.

**Figure 2:**
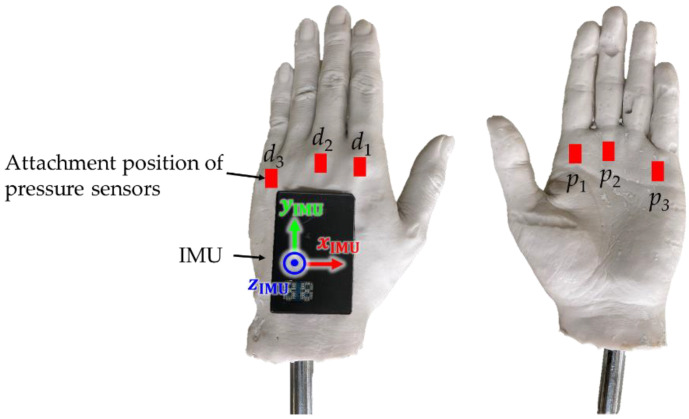
Attachment position of pressure sensors and IMU.

To investigate the effect of mounting an IMU on $F_{R}$, a hand model, waterproof IMU (SS-WP-HMA16G15, Sports Sensing Co. Ltd., Fukuoka, Japan), and waterproof wired pressure sensor (PS05-KC, Kyowa Electronic Instruments Co. Ltd., Tokyo, Japan) were used in the experiment (Figure 1). The hand model was fabricated using silicone and molded using a man’s left hand. The shape of the hand model featured a partially abducted thumb and a slightly convex dorsal side. Three pressure sensors and an IMU were attached to the dorsal side of the hand model, and the other three pressure sensors were attached to the palm side, as shown in Figure 2. Double-sided tape was used to mount these sensors, as in a previous study [18].

**Figure 3:**
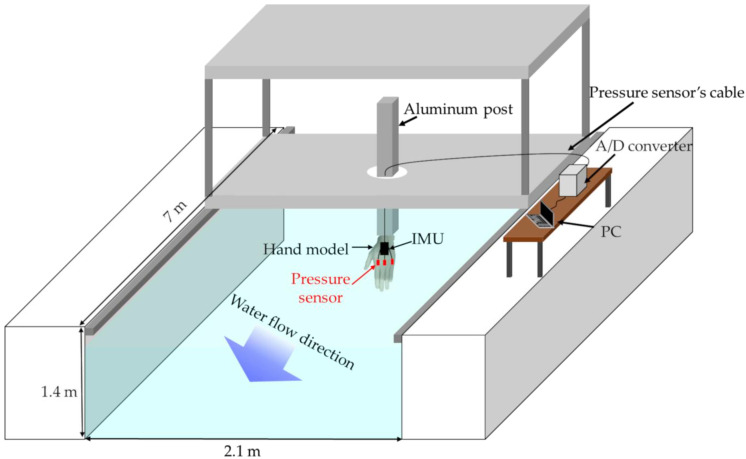
Schematic diagram showing experimental environment for Experiment 1.

The measurement was performed in a water flume with high reproducibility of the flow velocity. Figure 3 shows a schematic illustration of the experiment. The water temperature was 27 °C during the experiment. The hand model was fixed to an aluminum post, with the fingertips facing the bottom of the pool. The hand model was submerged approximately 0.3 m below the water surface. The pressure around the hand model was measured while the flow velocity and direction of the hand model were changed. This experiment was performed at two different flow velocities (1.2 and 1.6 m·s^−1^). The flow velocity was measured using a flow velocity meter (VR-301, KENEK Co., Ltd., Tokyo, Japan). The orientation of the hand model toward the water flow was changed manually. The water flow direction from the little finger side was defined as 0°, that perpendicular to the palm was defined as 90°, and that from thumb side was defined 180°; these directions changed every 30°. Measurements were first performed using the IMU and pressure sensors attached (referred to herein as “with the IMU”); subsequently, the IMU was removed and only the pressure sensors were attached (referred to herein as “without the IMU”). The measurements were performed for 10 s at each velocity and water flow direction, and were repeated 10 times. Pressure was recorded at a sampling rate of 200 Hz. The signal output from the pressure sensors was recorded on a personal computer (PC) using an A/D converter (EDX-100A, Kyowa Electronic Instruments Co. Ltd., Tokyo, Japan). Prior to the measurement, all the pressure sensors were confirmed to indicate a less-than-2.5% error toward theoretical hydrostatic pressures when submerged into calm water.

#### 2.1.2. Data Processing and Statistical Analysis

All the data were analyzed using numerical analysis software (MATLAB R2020a, MathWorks Inc., Natick, MA, USA). The pressure data were smoothed using a low-pass Butterworth digital filter. Based on residual analysis, the cutoff frequency for the pressure data was determined to be 20 Hz [31].

**Figure 4:**
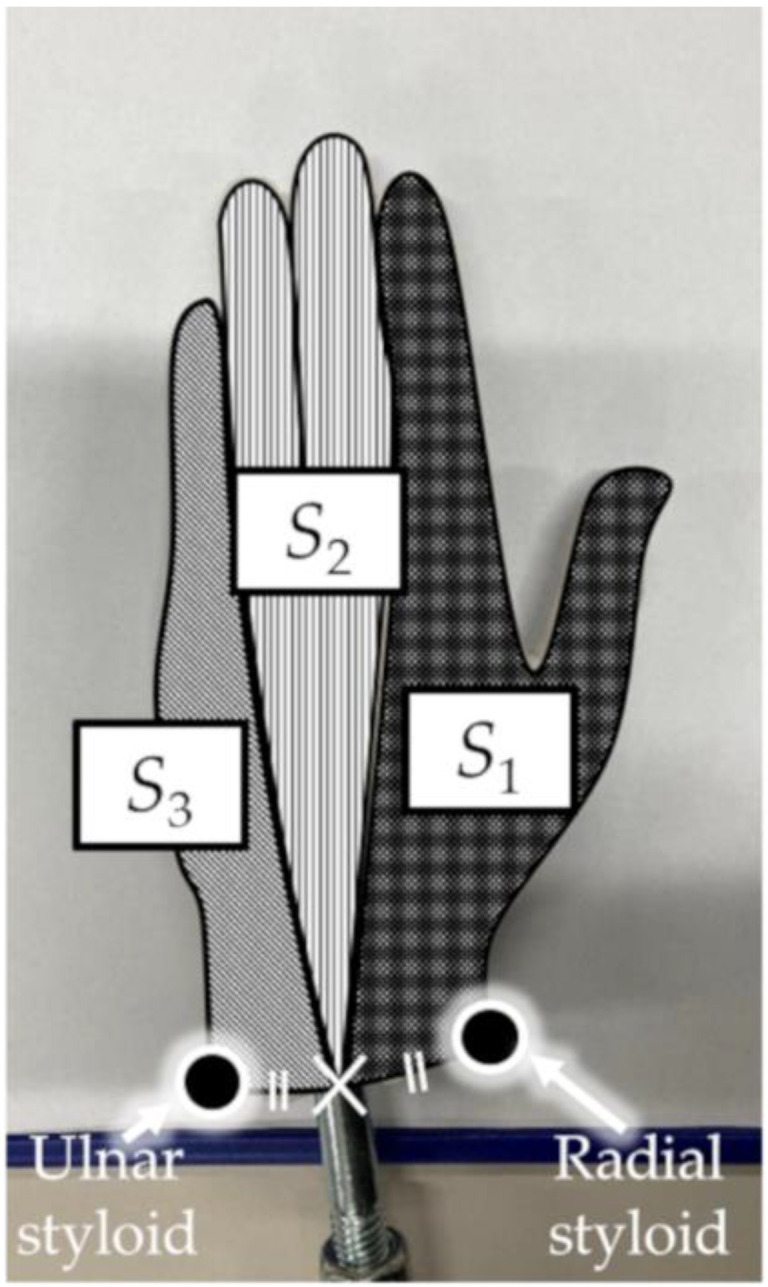
Segmentation of hand model area based on the radial styloid, ulnar styloid, and midpoint of two points.

The $F_{R}$ acting on the hand model was obtained by multiplying the hand model area (m^2^) by the pressure difference (N·m^−2^) between the palm (*p*_1_, *p*_2_, and *p*_3_) and dorsal (*d*_1_, *d*_2_, and *d*_3_) sides of the hand model [18]. The hand model area was measured by dividing the hand plane into three components (*S*_1_, *S*_2_, and *S*_3_) from photographs of the hand model using image-processing software (ImageJ, NIH, Bethesda, MD, USA) (see Figure 4). Subsequently, $F_{R}$ was calculated as the product of the pressure difference and the hand model area in each hand plane, followed by their summation, as shown in Equation (1).

(1) $$F_{R}=\sum_{i=1}^{3}S_{i}\left(p_{i}-d_{i}\right)\;$$

All the statistical analyses were performed using SPSS Statistics (version 25.0; IBM Corporation, Armonk, NY, USA). The mean values for 10 s of pressure and $F_{R}$ were calculated as the representative values for each condition (flow velocity and water flow direction). A paired *t*-test was performed to examine the differences in the pressure and $F_{R}$ with and without the IMU. The significance level was set to less than 5%. The effect size *d* was calculated using Cohen’s method [32]. The effect size magnitude was assessed using the thresholds provided by Cohen [33], i.e., *d* < 0.2 = “negligible”, *d* < 0.5 = “small”, and *d* < 0.8 = “medium”; otherwise, “large” is indicated.

### 2.2. Experiment 2: Propulsive Force Estimation

Five healthy male collegiate swimmers (age: 21 ± 1 years; height: 1.76 ± 0.04 m; mass: 70.0 ± 4.5 kg; hand area: 0.017 ± 0.001 m^2^) participated in this experiment. This study was reviewed and approved by the Ethics Committee of the institution to which the author belongs (Approval No. 11–34). All the participants voluntarily participated in this experiment after receiving an explanation regarding the purpose of the experiment, the details of the trial, and the risks associated with it. After the participants confirmed their complete understanding of the explanation, they submitted a consent form for participation in this experiment.

#### 2.2.1. Experimental Design

**Figure 5:**
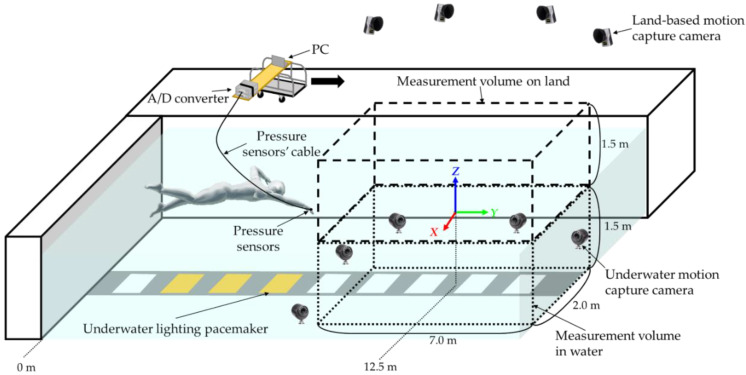
Schematic diagram showing experimental environment.

Figure 5 shows a schematic illustration of the experimental setup. The experiment was conducted in an indoor pool (length: 50 m; width: 25 m; depth: 2.0 m; water temperature: 27.5 °C). The participants performed 25 m of front crawl swimming twice at each of nine different swimming speeds from 0.8 to 1.6 m·s^−1^. The swimming speed was indicated to the participants via an underwater lighting pacemaker (Swimming Pace Maker System, Takei Scientific Instruments Co. Ltd., Niigata, Japan) installed at the bottom of the pool. Prior to each measurement, the participants stood in a position with both arms directed toward propulsion above the water and their palms facing down and parallel to the water surface. The participants began swimming by performing a wall push-off at the supervisor’s discretion. The rest time between each test was at least 1 min, where the rest time was longer in the latter half of the test when the swimming speed was higher. When the measurement could not be performed precisely, the same measurement was repeated.

To measure the pressure around the hands, six wired pressure sensors were attached to the palm and dorsal sides of both hands using double-sided tape in the same positions, as shown in Figure 2. The pressure was recorded at a sampling rate of 200 Hz. The signal output from the pressure sensors was recorded on a PC using an A/D converter (EDX-100A, Kyowa Electronic Instruments Co. Ltd., Tokyo, Japan). The PC and A/D converters were mounted on a push cart such that they propagated along with the swimmers.

**Figure 6:**
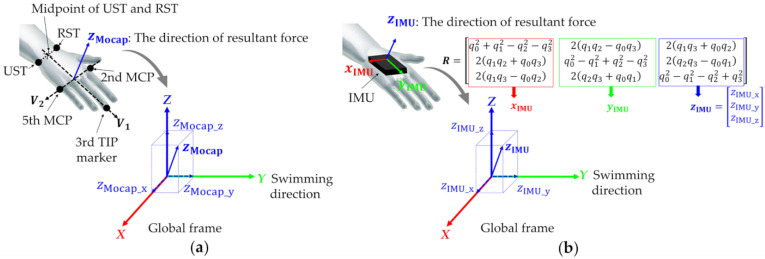
(**a**) Schematic diagram showing calculation of hand’s orientation using underwater motion-capture system. $z_{Mocap}$ (hand’s orientation) was calculated from the coordinates of five reflective markers on the hand (black dots). $z_{\mathrm{Mocap}\_y}$ represents the component of propulsive direction. (**b**) Schematic diagram showing estimation of hand’s orientation using IMU. Third column components of ***R*** represent $z_{IMU}$ direction in global frame. $z_{\mathrm{IMU}\_y}$ represents component of propulsive direction.

For motion-capture analysis, five landmark points were specified for each hand, similar to the procedure in [18]. Four 19 mm reflective markers were attached to the second and fifth metacarpophalangeal joints (hereinafter denoted as second MCP and fifth MCP, respectively), radial styloid (RST), and ulnar styloid (UST) using strong magnets and elastic medical cotton tape (Figure 6). Reflective tape was wrapped around the tip of the third finger, which was defined as the third TIP marker. Fourteen underwater cameras (Oqus3+ underwater, Qualisys, Göteborg, Sweden) and ten land-based cameras (Oqus3+, Qualisys, Göteborg, Sweden) were used for the experiment. The three-dimensional coordinates of the markers were recorded using motion-capture software (Qualisys Track Manager 2.15, Qualisys, Göteborg, Sweden) at a sampling rate of 200 Hz. The origin of the underwater motion-capture system was set at the water surface and 12.5 m away from the start. The transverse direction of the global frame was defined as the *X*-axis (right direction positive), the swimming direction as the *Y*-axis (forward positive), and vertical direction as the *Z*-axis (upward positive). The measurement volume was 2.0 m (*X*-axis) × 7.0 m (*Y*-axis) × 3.0 m (*Z*-axis), i.e., 1.5 m underwater and 1.5 m above the water surface. The measurement volume was set 9.0 m away from the pool wall. The accuracy within the analysis range after calibration was obtained using a 600.1 mm rod, and the errors on land and in water were confirmed to be 2.3 and 1.9 mm, respectively.

Waterproof IMUs were mounted on the dorsal side of each hand with double-sided tape and elastic medical cotton tape. The IMUs were mounted such that the $y_{IMU}$ direction coincided with the direction from the midpoint of the UST and RST to the third TIP marker (Figure 6b). The IMUs were recorded in the on-board memory at a sampling frequency of 200 Hz. Start signals from the IMU system were recorded in the underwater motion-capture system and A/D converter simultaneously, and then, used for data synchronization in the post-process.

#### 2.2.2. Data Processing

The underwater stroke phase was defined using the third TIP marker as the duration from entry into the water to exit from the water. In this study, one or two stroke cycles performed in the motion-capture area were analyzed, and the propulsive force during the underwater stroke phase was estimated. All the data were analyzed using numerical analysis software (MATLAB R2020a, MathWorks Inc., Natick, MA, USA). The coordinates and pressure data were smoothed using a low-pass Butterworth digital filter. Based on residual analysis [31], the cutoff frequencies were determined to be 6 and 20 Hz for the coordinate and pressure data [18], respectively.

The $F_{R}$ of each hand was calculated by multiplying the pressure difference with the hand area (Equation (1)). The hand area was divided based on the UST, RST, and the midpoints of the UST and RST.

To estimate the propulsive force generated by the hands, hand orientations were obtained using an underwater motion-capture system and an IMU. For the method using an underwater motion-capture system, the hand orientations were calculated using the coordinates of markers attached to the hands, based on procedures reported previously [4,18] (Figure 6a). The vector from the midpoint of the UST and RST to the third TIP marker is defined as ***V*_1_**. For the right hand, the vector from the second to the fifth MCP is defined as ***V*_2_.** By contrast, for the left hand, the vector from the fifth to the second MCP is defined as ***V*_2_**. The hand orientations, $z_{Mocap}={\left[z_{\mathrm{Mocap}\_x},\;\;z_{\mathrm{Mocap}\_y},\;\;z_{\mathrm{Mocap}\_z}\right]}^{T}$**_,_** are unit vectors calculated by the cross-product of ***V*_2_** and ***V*_1_** and normalized by its own norm. Therefore, the propulsive force estimated using the underwater motion-capture system and pressure sensors ($F_{\mathrm{Mocap}}$) can be determined using Equation (2).

(2) $$F_{\mathrm{Mocap}}=F_{R}\cdot z_{\mathrm{Mocap}\_y}$$

For the IMU, the hand orientations were obtained using the sensor fusion algorithm proposed by Madgwick (the divergence rate: *β* = 0.033) [34]. Researchers have reported that the magnetometer may be disturbed by ferromagnetic objects, such as water pumps under the pool [35,36]. Hence, the sensor orientation was obtained by processing the raw acceleration and angular velocity. The orientation was expressed by a quaternion comprising a scalar component, $q_{0}$, and vector components, $q_{1}$ to $q_{3}$. The representation of the hand orientation was transformed from a quaternion to a rotation matrix (***R***) using Equation (3), as follows:

(3) $$R=\left[\begin{array}{ccc} q_{0}^{2}+q_{1}^{2}-q_{2}^{2}-q_{3}^{2} & 2\left(q_{1}q_{2}-q_{0}q_{3}\right) & 2\left(q_{1}q_{3}+q_{0}q_{2}\right)\\ 2\left(q_{1}q_{2}+q_{0}q_{3}\right) & q_{0}^{2}-q_{1}^{2}+q_{2}^{2}-q_{3}^{2} & 2\left(q_{2}q_{3}-q_{0}q_{1}\right)\\ 2\left(q_{1}q_{3}-q_{0}q_{2}\right) & 2\left(q_{2}q_{3}+q_{0}q_{1}\right) & q_{0}^{2}-q_{1}^{2}-q_{2}^{2}+q_{3}^{2} \end{array}\right]$$

In the matrix above, each column of ***R*** represents the axis direction of the sensor frame ($x_{IMU}$, $y_{IMU}$, and $z_{IMU}$) in the global frame (Figure 6b). $z_{IMU}$ is assumed to be orthogonal to the palm, and the Y component of $z_{IMU}$ ($z_{\mathrm{IMU}\_y}$) is used as the swimming direction for estimating the propulsive force (Equation (4)).

(4) $$F_{\mathrm{IMU}}=F_{R}\cdot z_{\mathrm{IMU}\_y}=F_{R}\cdot 2\left(q_{2}q_{3}-q_{0}q_{1}\right)$$

#### 2.2.3. Statistical Analysis

$F_{\mathrm{Mocap}}$ and $F_{\mathrm{IMU}}$ were compared using data from five participants, which comprised 297 strokes. The agreement between the two methods was evaluated based on the mean values and waveforms of $F_{\mathrm{Mocap}}$ and $F_{\mathrm{IMU}}$. The mean values of $F_{\mathrm{Mocap}}$ and $F_{\mathrm{IMU}}$ during the underwater stroke phase are denoted as $\bar{F_{\mathrm{Mocap}}}$ and $\bar{F_{\mathrm{IMU}}}$, respectively. Agreement between $\bar{F_{\mathrm{Mocap}}}$ and $\bar{F_{\mathrm{IMU}}}$ was assessed using the intraclass correlation coefficient (ICC_(2,1)_) and Bland–Altman analysis [37]. The waveform similarity between the $F_{\mathrm{Mocap}}$ and $F_{\mathrm{IMU}}$ waveforms was assessed based on the coefficient of multiple correlation (CMC).

ICC_(2,1)_ was calculated using a two-way random-effects model. Values from 0.75 to 0.90 have been suggested as acceptable values; in fact, they should exceed 0.90 for most clinical measurements [38,39]. In this study, ICC_(2,1)_ values less than 0.75 were defined as low agreement, 0.75 to 0.90 as medium agreement, and greater than 0.90 as good agreement, based on [38].

Bland–Altman analysis was performed to confirm fixed and proportional biases. The fixed bias was examined using a one-sample *t*-test (test value = 0) of the difference between $\bar{F_{\mathrm{Mocap}}}$ and $\bar{F_{\mathrm{IMU}}}$ (Diff). The proportional bias was examined using the Pearson product-moment coefficient of correlation *r* between the mean and Diff. The significance level was set to less than 5%. The effect size *d* was calculated and assessed using the same methods and thresholds as in Experiment 1. The standard deviation of the difference between $\bar{F_{\mathrm{Mocap}}}$ and $\bar{F_{\mathrm{IMU}}}$ was corrected based on the mean square and denoted as ${\mathrm{SD}}_{\mathrm{Diff}}$. The 95% limit of agreement (95% LOA) was calculated by multiplying ${\mathrm{SD}}_{\mathrm{Diff}}$ by ±1.96 based on [40]. In addition, the upper and lower limits of the confidence interval for the 95% LOA were calculated using Equations (5) and (6) [37,40], respectively.

(5) $$\mathrm{Upper}\;\mathrm{limits}=\left(\bar{\mathrm{Diff}}+1.96\cdot {\mathrm{SD}}_{\mathrm{Diff}}\right)-t\sqrt{3\cdot {\mathrm{SD}}_{\mathrm{Diff}}{}^{2}/n}$$

(6) $$\mathrm{Lower}\;\mathrm{limits}=\left(\bar{\mathrm{Diff}}-1.96\cdot {\mathrm{SD}}_{\mathrm{Diff}}\right)+t\sqrt{3\cdot {\mathrm{SD}}_{\mathrm{Diff}}{}^{2}/n}$$

where $\bar{\mathrm{Diff}}$ is the mean of Diff, *t* is the *t*-value with a two-sided probability of 5% in the *t*-distribution with *n*−1 degrees of freedom, and *n* is the sample size (*n* = 297).

The CMC was used to evaluate the waveform similarity, and it was calculated using the formula provided in [41]. The CMC values were defined as having poor similarity (0–0.64), moderate similarity (0.65–0.74), good similarity (0.75–0.84), very good similarity (0.85–0.94), or excellent similarity (0.95–1), based on [42].

## 3. Results

### 3.1. Effect of Mounted IMU on Hand Model

**Figure 7:**
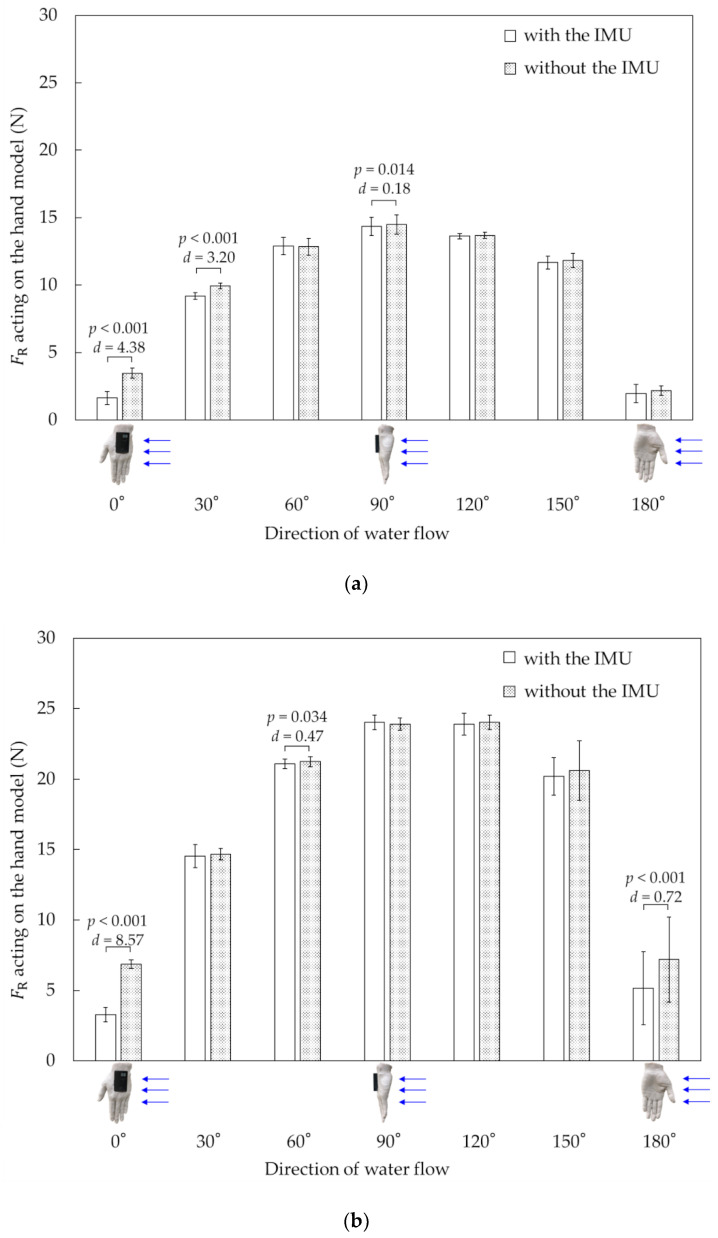
Variation in resultant force with and without IMU with variations in flow velocity and water flow direction. (**a**) Flow velocity = 1.2 m·s^−1^; (**b**) flow velocity = 1.6 m·s^−1^.

Figure 7 shows the differences in $F_{R}$ with and without the IMU at two different flow velocities. At 1.2 m·s^−1^, significant differences were indicated between the cases with and without the IMU at 0° (*t* = −11.99; *p* < 0.001; *d* = 4.38), 30° (*t* = −7.60; *p* = < 0.001; *d* = 3.20), and 90° (*t* = −3.05; *p* = 0.014; *d* = 0.18). Meanwhile, at 1.6 m·s^−1^, significant differences were indicated at 0° (*t* = −32.60; *p* < 0.001; *d* = 8.57), 60° (*t* = −2.50; *p* = 0.034; *d* = 0.47), and 180° (*t* = −9.46; *p* < 0.001; *d* = 0.72). 

The most significant difference was observed at 0° for both flow velocities. Therefore, the pressure at 0° was prioritized in this study. Table 1 and Table 2 show the pressures on the palm and dorsal sides of the hand model at 0°. Significant differences were indicated between the pressure recorded with and without the IMU in all six pressure sensor positions. In particular, the effect size of *d*_3_ was the largest in the six pressure sensors for both flow velocities (1.2 m·s^−1^, *d* = 9.03; 1.6 m·s^−1^, *d* = 8.81). This suggests that when water flows from the little finger side, the hand-mounted IMU affects the pressure at *d*_3_.

### 3.2. Comparison of Two Propulsive Force Estimation Methods 

**Figure 8:**
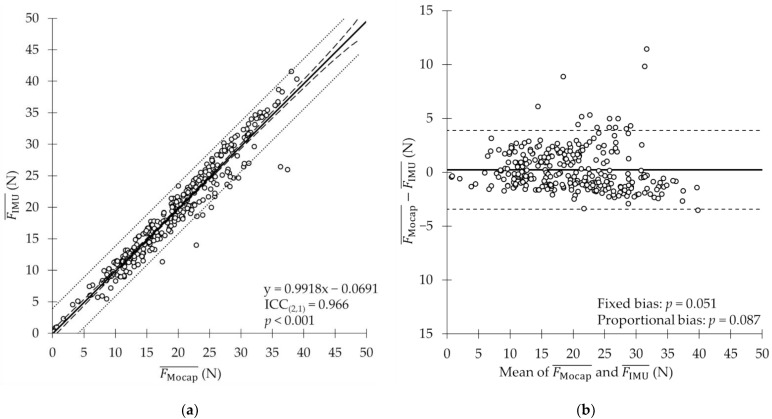
(**a**) Comparison of $\bar{F_{\mathrm{Mocap}}}$ and $\bar{F_{\mathrm{IMU}}}$. Solid line represents regression line, dashed line represents confidence interval of regression line, and dotted line represents prediction interval. (**b**) Results of Bland–Altman analysis of mean propulsive force. Dashed line represents upper and lower limits of confidence interval for 95% LOA.

A comparison of the mean values shows that the $\bar{F_{\mathrm{Mocap}}}$ and $\bar{F_{\mathrm{IMU}}}$ of all 297 strokes in Experiment 2 were 19.59 ± 7.66 and 19.36 ± 7.86 N, respectively. $\bar{\mathrm{Diff}}$ was 0.23 N (relative error: 0.4%), and ${\mathrm{SD}}_{\mathrm{Diff}}$ was ±2.08 N. The ICC_(2,1)_ between $\bar{F_{\mathrm{Mocap}}}$ and $\bar{F_{\mathrm{IMU}}}$ was 0.966 (95% confidence interval: 0.958–0.973, *p* < 0.001), which indicates good agreement between them (Figure 8a). The Bland–Altman analysis showed neither fixed (*p* = 0.051) nor proportional (*p* = 0.087) biases between $\bar{F_{\mathrm{Mocap}}}$ and $\bar{F_{\mathrm{IMU}}}$ (Figure 8b).

**Figure 9:**
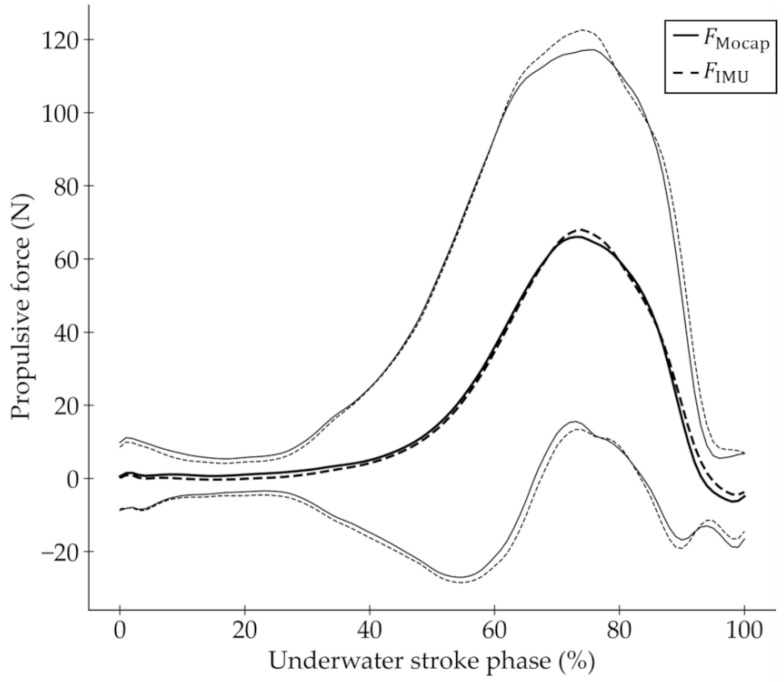
Ensemble means and standard deviations (SDs) for $F_{\mathrm{Mocap}}$ and $F_{\mathrm{IMU}}$ for all strokes. Ensemble means are shown as thick solid line ($F_{\mathrm{Mocap}}$) and thick dashed line ($F_{\mathrm{IMU}}$). Thin solid and thin dashed lines represent ±1.96 SDs of $F_{\mathrm{Mocap}}$ and $F_{\mathrm{IMU}}$, respectively.

Figure 9 shows the ensemble means ±1.96 standard deviations (SDs) for the $F_{\mathrm{Mocap}}$ and $F_{\mathrm{IMU}}$ of all the strokes. The waveform of $F_{\mathrm{Mocap}}$ is similar to that of $F_{\mathrm{IMU}}$ (CMC = 0.99).

## 4. Discussion

### 4.1. Effect of Hand-Mounted IMU on $F_{R}$ Acting on Hand

**Figure 10:**
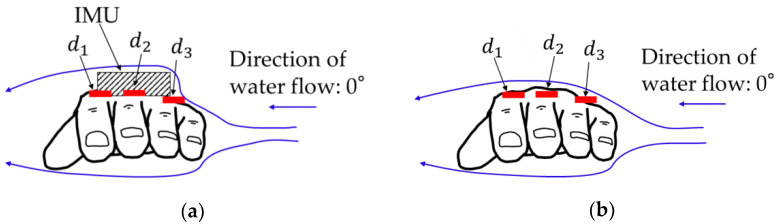
Conceptual diagram showing difference in water flow around hand with (**a**) and without (**b**) the IMU.

In Experiment 1, the effect of a hand-mounted IMU on the $F_{R}$ acting on the hand was investigated by changing the flow velocity and water flow direction relative to the hand. The results showed that the hand-mounted IMU affected $F_{R}$; however, the effect of the hand-mounted IMU varied depending on the water flow direction. In this study, the water flow direction from the little finger side was set to 0°, and the water flow direction from the thumb side was set to 180°. From 60° to 150°, the effect of the hand-mounted IMU on $F_{R}$ was negligible or small (Figure 7). For both flow velocities, decrements in the resultant force were observed at the water flow direction of 0° from the hand-mounted IMU, and significant pressure differences were observed between the cases with and without the IMU at each pressure sensor position (Table 1 and Table 2). The difference in pressure is attributable to the change in the water flow around the hand model owing to the hand-mounted IMU. Figure 10 shows a conceptual diagram of the difference in water flow with and without the IMU when the water flow direction was 0°. The pressure was predicted to increase owing to the decreased flow velocity due to flow obstruction by the hand-mounted IMU. In this experiment, the pressure at *d*_3_ without the IMU was lower than that with the IMU, and the effect of the hand-mounted IMU on the pressure at *d*_3_ was the most prominent among the pressure sensor positions investigated. Therefore, mounting the IMU on the dorsal side of the hand may change the flow and pressure, particularly on the dorsal hand side. However, assuming front crawl swimming, the water flowing from the little finger side was at the end of the underwater stroke phase, immediately before the hand exited the water. The phase immediately before the hand exited the water was confirmed to be short throughout the entire underwater stroke phase [43]. In addition, swimmers typically maintain the angle of attack of their hands at 50°–70° in three swimming styles: front crawl, backstroke, and butterfly [1,44]. Therefore, the effect of using an IMU in front crawl swimming on $F_{R}$ would be insignificant.

We examined the effect of a hand-mounted IMU on $F_{R}$ at two different velocities using a hand model fixed to an aluminum post. However, many researchers have indicated the importance of considering unsteady flow and motion in swimming research because swimming is a complex motion with repeated acceleration and deceleration [10,11,12,13,14]. Therefore, the effects of hand-mounted IMUs on $F_{R}$ at higher velocities and in actual swimming motion should be investigated more comprehensively in future studies. The smaller size of the IMU (especially the thinner IMU) will reduce the obstruction of water flow on swimmers’ hands. However, the influence of sensors on swimming never disappears compared to bare hands. A more detailed investigation is possible through flow analysis, such as a simulation or particle image velocimetry.

### 4.2. Agreement between Propulsive Force Estimation Using IMU and Underwater Motion-Capture System

In Experiment 2, the propulsive force estimated via a novel method using an IMU and pressure sensors ($F_{\mathrm{IMU}}$) was compared with the propulsive force estimated using a method that utilizes an underwater motion-capture system ($F_{\mathrm{Mocap}}$). A high ICC_(2,1)_ was obtained, and no fixed or proportional biases were indicated between $\bar{F_{\mathrm{Mocap}}}$ and $\bar{F_{\mathrm{IMU}}}$. The similarity evaluation of the waveforms of both propulsive forces showed a CMC of 0.99, which reflects high waveform similarity. These results indicate that the IMU and pressure sensors can be used to evaluate the propulsive force generated by the swimmer’s hands, similar to the method using an underwater motion-capture system and pressure sensors. 

In land exercises, oscillations between the body and IMU (or reflective markers), i.e., soft-tissue artifacts, are often regarded as error factors for estimating orientation [36,45,46,47,48]. The effect of soft-tissue artifacts is predicted to be significant for explosive movements [36]. For example, in front crawl swimming, the effect of soft-tissue artifacts is predicted to be more prominent when the hands and feet enter the water, particularly at high swimming speeds. Furthermore, it has been reported that vortices are generated around the hand, and that the water flow becomes more complex and faster in the latter half of the underwater stroke phase when a large force is generated [13,14]. Therefore, the effect of the IMU and reflective marker oscillations on the estimation of hand orientation during swimming must be considered. This effect may be minimized, for example, by using smaller sensors. Furthermore, drift errors caused by long-period measurements were not considered because the measurements performed in this study were short. Nonetheless, further investigations are required.

Unlike underwater motion-capture systems, IMUs are not affected by bubbles when estimating hand orientation. Moreover, IMUs require less time for post-processing and incur a lower cost than underwater motion-capture systems. Hence, IMUs can be applied widely to coaching, e.g., the daily evaluation of swimmers’ performance. Using an IMU and pressure sensors to evaluate the propulsive force generated by the hands offers advantages that cannot be provided by underwater motion-capture systems. To render the proposed method more accessible for coaching, we believe that efforts should be undertaken to minimize the burden on swimmers, such as miniaturizing IMUs and eliminating pressure sensor cables by integrating IMUs with pressure sensors.

## 5. Conclusions

The aims of this study were (1) to verify the effect of a hand-mounted IMU on the $F_{R}$ acting on the hand, and (2) to propose a method of evaluating the propulsive force generated by the hands using IMUs and pressure sensors ($F_{\mathrm{IMU}}$); then, we compared the results with the value estimated using an underwater motion-capture system and pressure sensors ($F_{\mathrm{Mocap}}$). In Experiment 1, decrements in $F_{R}$ were observed in the water flowing from the little finger side by mounting an IMU at flow velocities of 1.2 and 1.6 m·s^−1^. Significant differences in pressure were indicated for the cases with and without the IMU at each pressure sensor position. In Experiment 2, the mean values of $F_{\mathrm{Mocap}}$ and $F_{\mathrm{IMU}}$ indicated good agreement and exhibited neither fixed nor proportional biases. Additionally, the waveforms of $F_{\mathrm{Mocap}}$ and $F_{\mathrm{IMU}}$ were similar.

## Figures and Tables

**Table 1:** Comparison of pressure on hand model between cases with and without the IMU based on 0° and 1.2 m·s^−1^.

Pressure Sensor’s Position	Pressure without the IMU (kPa)	Pressure with the IMU (kPa)	*t* Value (*p* Value)	Cohen’s *d*
*p*_1_	−0.63 ± 0.02	−0.70 ± 0.02	−7.30 (<0.001)	2.94
*p*_2_	−0.73 ± 0.02	−0.77 ± 0.01	−8.59 (<0.001)	2.04
*p*_3_	−0.69 ± 0.04	−0.74 ± 0.03	−3.97 (<0.001)	1.31
*d*_1_	−0.85 ± 0.03	−0.89 ± 0.02	−11.21 (<0.001)	1.45
*d*_2_	−0.99 ± 0.02	−0.95 ± 0.04	6.26 (<0.001)	1.45
*d*_3_	−0.98 ± 0.04	−0.63 ± 0.03	22.38 (<0.001)	9.03

**Table 2:** Comparison of pressure on hand model between cases with and without the IMU based on 0° and 1.6 m·s^−1^.

Pressure Sensor’s Position	Pressure without the IMU (kPa)	Pressure with the IMU (kPa)	*t* Value (*p* Value)	Cohen’s *d*
*p*_1_	−0.65 ± 0.05	−0.75 ± 0.04	−18.61 (<0.001)	2.14
*p*_2_	−0.90 ± 0.06	−1.01 ± 0.08	−7.93 (<0.001)	1.51
*p*_3_	−0.70 ± 0.08	−0.79 ± 0.08	−23.67 (<0.001)	1.15
*d*_1_	−1.09 ± 0.09	−1.12 ± 0.09	−6.00 (<0.001)	0.37
*d*_2_	−1.25 ± 0.09	−1.14 ± 0.10	13.96 (<0.001)	1.19
*d*_3_	−1.48 ± 0.07	−0.92 ± 0.06	35.24 (<0.001)	8.81

## Data Availability

The data analyzed in this manuscript can be made available from the corresponding author upon reasonable request.
